# Cord Blood Vα24-Vβ11^+^ Natural Killer T Cells Display a Th2-Chemokine Receptor Profile and Cytokine Responses

**DOI:** 10.1371/journal.pone.0015714

**Published:** 2011-01-31

**Authors:** Susanne Harner, Elfriede Noessner, Korinna Nadas, Anke Leumann-Runge, Matthias Schiemann, Fabienne L. Faber, Joachim Heinrich, Susanne Krauss-Etschmann

**Affiliations:** 1 Comprehensive Pneumology Center, Ludwig-Maximilians University Hospital and Helmholtz Zentrum München, Großhadern, Germany; 2 Institute of Molecular Immunology, Helmholtz Zentrum München, German Research Center for Environmental Health, Großhadern, Germany; 3 Institute of Medical Microbiology, Immunology and Hygiene, Technical University Munich, Munich, Germany; 4 Clinical Cooperation Group “Immune-Monitoring”, Helmholtz Zentrum München, German Research Center for Environmental Health, Neuherberg, Germany; 5 Children's Hospital of the Ludwig-Maximilians University, Munich, Germany; 6 Institute of Epidemiology, Helmholtz Zentrum Munich, German Research Center for Environmental Health, Neuherberg, Germany; New York University, United States of America

## Abstract

**Background:**

The fetal immune system is characterized by a Th2 bias but it is unclear how the Th2 predominance is established. Natural killer T (NKT) cells are a rare subset of T cells with immune regulatory functions and are already activated *in utero*. To test the hypothesis that NKT cells are part of the regulatory network that sets the fetal Th2 predominance, percentages of Vα24^+^Vβ11^+^ NKT cells expressing Th1/Th2-related chemokine receptors (CKR) were assessed in cord blood. Furthermore, IL-4 and IFN-γ secreting NKT cells were quantified within the single CKR^+^ subsets.

**Results:**

Cord blood NKT cells expressed the Th2-related CCR4 and CCR8 at significantly higher frequencies compared to peripheral blood NKT cells from adults, while CXCR3^+^ and CCR5^+^ cord blood NKT cells (Th1-related) were present at lower percentages. Within CD4^neg^CD8^neg^ (DN) NKT cells, the frequency of IL-4 producing NKT cells was significantly higher in cord blood, while frequencies of IFN-γ secreting DN NKT cells tended to be lower. A further subanalysis showed that the higher percentage of IL-4 secreting DN NKT cells was restricted to CCR3^+^, CCR4^+^, CCR5^+^, CCR6^+^, CCR7^+^, CCR8^+^ and CXCR4^+^ DN subsets in cord blood. This resulted in significantly decreased IFN-γ /IL-4 ratios of CCR3^+^, CCR6^+^ and CCR8^+^ cord blood DN NKT cells. Sequencing of VA24AJ18 T cell receptor (TCR) transcripts in sorted cord blood Vα24Vβ11 cells confirmed the invariant TCR alpha-chain ruling out the possibility that these cells represent an unusual subset of conventional T cells.

**Conclusions:**

Despite the heterogeneity of cord blood NKT cells, we observed a clear Th2-bias at the phenotypic and functional level which was mainly found in the DN subset. Therefore, we speculate that NKT cells are important for the initiation and control of the fetal Th2 environment which is needed to maintain tolerance towards self-antigens as well as non-inherited maternal antigens.

## Introduction

The fetus is exposed to a unique environment and needs to maintain tolerance both towards non-inherited maternal antigens and self-antigens that arise during its development. During pregnancy both maternal and fetal immunity are characterized by a Th2-bias. This promotes reciprocal materno-fetal tolerance and ensures fetal survival [1], [2]. However, it is unclear how the fetal Th2-predominance is established.

Natural Killer T (NKT) cells are a rare subset of highly conserved T cells that are already activated *in utero*. They are characterized by the simultaneous expression of a T cell receptor (TCR) and the NK cell marker CD161 (NKR-P1A) which places them at the interface of innate and adaptive immunity. Most human peripheral blood NKT cells are CD4^+^ or CD4^−^CD8^−^ double negative (DN), while CD8^+^ NKT cells are less frequent [3], [4]. They further display an activated memory phenotype as shown by surface expression of CD25^hi^, CD45RO^hi^, CD45RB^low^ and CD62L^low^ [5], [6], [7]. A hallmark of NKT cells is their highly restricted TCR alpha repertoire, with constant expression of the T cell receptor alpha variable chain 24 (TCRAV24) gene segment that rearranges almost exclusively with T cell receptor alpha chain joining region 18 (TCRAJ18), which result in the pairing of T cell receptor alpha chain 24 (Vα24) and T cell receptor beta chain 11 (Vβ11). Furthermore, the N-region of the TCRA chain does not contain inserted nucleotides [8], [9] although rare exceptions from this rule have been described [8], [10], [11]. The TCR repertoire of NKT cells is further restricted by the preferential pairing of TCRAV24-AJ18 with polyclonal TCRBV11 transcripts that use diverse TCRBJ gene segments and complementarity determining regions 3 (CDR3). Despite this strongly limited TCR repertoire NKT cells regulate a broad range of immune responses including the maintenance of tolerance [12] or the defense against bacterial infections [13], [14], [15]. Although the mechanistic basis for the regulatory function of NKT cells is incompletely understood, it is mediated at least partly by the unique ability of NKT cells to produce Th1 and Th2 cytokines rapidly and in high concentrations [16], [17], [18], [19].

NKT cells are also found in cord blood [5], [6] where they already express the surface markers associated with activation. This suggests that NKT cells might regulate immune functions as early as in fetal life. Therefore, we hypothesized that cord blood NKT cells possess unique regulatory functions that first, are distinct from those of adult blood and second help to set the Th2 predominance.

To test this hypothesis, we performed a comprehensive analysis of homeostatic and inflammatory chemokine receptors expressed on cord blood NKT cells as compared to peripheral blood NKT cells from adults and further determined the frequencies of Interferon-γ (IFN-γ) and Interleukin-4 (IL-4) secreting NKT cells within these subsets.

## Methods

### Subjects

Peripheral blood was drawn from 28 healthy, non-smoking adult volunteers without allergic disease (median age 27 yrs; range 23–45; 12 males, 13 females). In addition, a peripheral blood count was performed in EDTA-blood to exclude infection and samples with low lymphocyte counts.

Cord blood was obtained from 29 healthy, vaginally delivered term newborns. Newborns who were small or large for gestational age, with APGAR 10 min ≤7, obvious malformations or with clinically suspected infection or maternal signs of infection sub partu (fever>38.5°, leucocytosis>15.000/µl, CRP>20mg/dl) were excluded a priori. To control for contamination with maternal blood only cord serum IgA was determined (below detection limit of 4.2 mg/dl). A cord blood count was performed in EDTA-blood to further exclude anemia and/or infection. Written informed consent was obtained from the mothers. The study protocol was approved by the local Ethics Committee of the Bavarian College of Physicians. All clinical investigations have been conducted according to the principles of the Declaration of Helsinki.

### Flow cytometry

To assess the chemokine receptors on the surface of NKT cells four-color flow cytometry (FACS Calibur, Becton-Dickinson, Heidelberg, Germany) was performed on freshly isolated mononuclear cells from cord blood and peripheral blood from adults. NKT cells were detected with mouse Vα24-FITC, biotinylated mouse Vβ11 (Beckmann Coulter, Krefeld, Germany) and strepavidin-allophycocyanin (APC) (Becton-Dickinson). For chemokine receptor analysis mouse anti-CCR1, -CCR2, -CCR6, -CCR7, -CCR8, -CCR9, -CXCR3, -CXCR4, -CXCR5, -CXCR6 coupled to phycoerythrin (PE) (all from R&D, Wiesbaden), anti-CCR4-PE, anti-CCR5-PE, and anti-CD45RO-PE (Becton-Dickinson) were used in combination with mouse CD4-phycoerythrin-cyanine 5 (PC5) or with CD8-PC5 (IQ-Products, Groningen). For the assessment of intracellular cytokines (see below) six color flow-cytometry (FACS CANTO, Becton-Dickinson) was performed using mouse anti-CD8 coupled to the tandem dye peridinin-chlorophyll protein-cyanine-dye5.5 (PerCP-Cy5.5), mouse CD4 APC-cyanine dye 7 (Cy7) (Becton Dickinson) and rat anti-CCR3, CCR8) or mouse CKR-antibodies coupled to APC (all R&D, Wiesbaden). Biotinylated mouse Vβ11 was detected with strepavidin-PE-Cy7. Color matched mouse IgG_1_, mouse IgG_2a_, mouse and rat IgG_2b_ were used as isotype controls. A median of 3.95×10^5^ and 7.92×10^5^ lymphocytes was acquired in adults and cord blood. To determine the percentages of Vα24^+^Vβ11^+^ positive NKT cells, lymphocytes were first gated according to their forward/side-scatter characteristics. A second gate was set on Vα24^+^Vβ11^+^ lymphocytes and the percentage of chemokine receptor positive cells was calculated within CD4^+^ and CD8^+^ NKT cell subsets, after defining a cutoff value according to the isotype controls. Data were analyzed with BD FACS DIVA Software version 5.0.3.; Becton Dickinson).

### Induction of intracellular cytokine production

Mononuclear cells were stimulated with phorbol myristate acetate (PMA) (10 ng/ml) and ionomycine (0.75 µg/ml) in the presence of 10 µg/ml brefeldin A for 3.5 hours (IL-4, IFN-γ, IL-13) or 24 h (IL-10) in VLE-RPMI 1640 followed by a fixation step for 10 min at room temperature with Cellfix (Becton Dickinson). After permeabilization with 0.5% saponin solution for 10 min at 4°C cells were stained with PE-conjugated monoclonal antibodies directed against the respective cytokines. To minimize intraobserver variations the cytokine data were analyzed two times at different occasions and mean values were calculated.

### N-region analysis of isolated NKT cells

NKT cells were highly enriched by FACS sorting (MoFlo XDP, Beckman Coulter, FL, USA) and processed immediately after sorting. Total mRNA was extracted using RNEasy MicroKit (QIAGEN, Hilden) according to the manufacturer's instruction with a QIA Cube (QIAGEN) and immediately reverse transcribed using random hexamer priming (Reverse Transcriptase Primer Mix, QIAGEN). Contamination with genomic DNA was excluded by RNA controls without reverse transcriptase in the cDNA synthesis reaction. AV24-AJ18 transcripts were amplified using AV24 5′-GAA CTG CAC TCT TCA ATG C-3′and AJ18 5′-TCC AAA GTA TAG CCT CCC CAG-3′ as forward and reverse primers (Metabion, Martinsried). PCRs were run on a TGradient (Biometra, Göttingen) for 3 min at 95°C, followed by 37 cycles (40 sec at 94°C, 40 sec at 59.7°C) and 1 min at 72°C using Taq-Polymerase (Roche, Penzberg). Amplicons were sequenced by MWG Eurofins (Martinsried, Munich, Germany).

### Statistical analysis

Due to the low frequency of NKT cells a normal distribution could not be expected. Therefore, Mann-Whitney *U* test was used to analyze differences between NKT cells from adult peripheral blood and cord blood. A probability of p≤0.05 was regarded as statistically significant. Statistical analyses were performed with Prism 5.0 (GraphPad Software, Inc. La Jolla, CA 92037 USA).

## Results

### Chemokine receptor expression patterns of cord blood Vα24^+^ Vβ11^+^ NKT cells

The frequency of cord blood NKT cells, defined as the Vα24^+^ Vβ11^+^ cells, within all lymphocytes ranged from 0.02–0.12% (median 0.05%) and tended to be higher than in peripheral NKT cells from adults (median 0.07%; range 0.01–0.23%). In cord blood, the percentage of CD4^+^ NKT within all Vα24^+^ Vβ11^+^ NKT cells was significantly higher as compared to peripheral blood from adults (median 89.7%, range 70.7–99.3% versus 17.8%, range 4.6–45.5%; p<0.0001), while CD8^+^ NKT cells did not differ between both groups (median 6.2% versus 5.3%). In a first step, we examined whether a panel of chemokine receptors is differentially expressed on cord blood versus peripheral NKT cells: cord blood NKT cells expressed the Th2-associated chemokine receptors CCR4 and CCR8 at significantly higher frequencies compared to NKT cells from adults, while the frequency of cord blood NKT cells bearing the Th1-associated CXCR3 or CCR5 was lower (significant for CCR5 only). Further, percentages of CCR7^+^, CCR9^+^, CXCR5^+^ and CXCR6^+^ NKT cells were significantly higher in cord blood. Percentages of CD45RO expressing NKT cells were lower in cord blood (**Table 1**).

**Table 1:** Percentages of chemokine receptor expressing Vα24^+^Vβ11^+^ NKT cells.

	Cord blood	Peripheral blood	p-value
**CCR1**	57.08^a^ (38.78–72.73)	54.58 (37.36–61.97)	0.48
**CCR2**	51.38 (32.30–58.73)	48.75 (28.57–63.69	0.97
**CCR4**	83.3 (71.8–90.4)	17.6 (7.3–37.6)	**<0.0001**
**CCR5**	47.28 (27.87–56.80)	79.78 (73.53–91.42)	**<0.0001**
**CCR6**	44.96 (41.36–63.21)	43.50 (9.23–55.00)	0.48
**CCR7**	90.50 (66.67–97.78)	43.67 (31.35–57.63)	**<0.0001**
**CCR8**	54.05 (32.00–77.78)	35.95 (11.02–55.05)	**0.03**
**CCR9**	10.68 (4.35–17.19)	4.98 (0.48–12.06)	**0.01**
**CXCR3**	83.09 (69.28–99.17)	94.09 (81.12–96.94)	0.13
**CXCR4**	86.27 (69.77–99.17)	84.30 (56.90–100.00)	0.60
**CXCR5**	40.00 (26.22–43.88)	31.48 (23.66–38.87)	**0.03**
**CXCR6**	54.29 (50.00–66.13)	48.28 (34.47–54.55)	**<0.01**
**CD45RO**	92.86 (88.75–97.20)	98.00 (94.83–100.00)	**<0.002**

a Data are shown as medians with (min-max.).

### Cytokine secretion within specific chemokine receptor expressing NKT cell subsets

**Figure 1:**
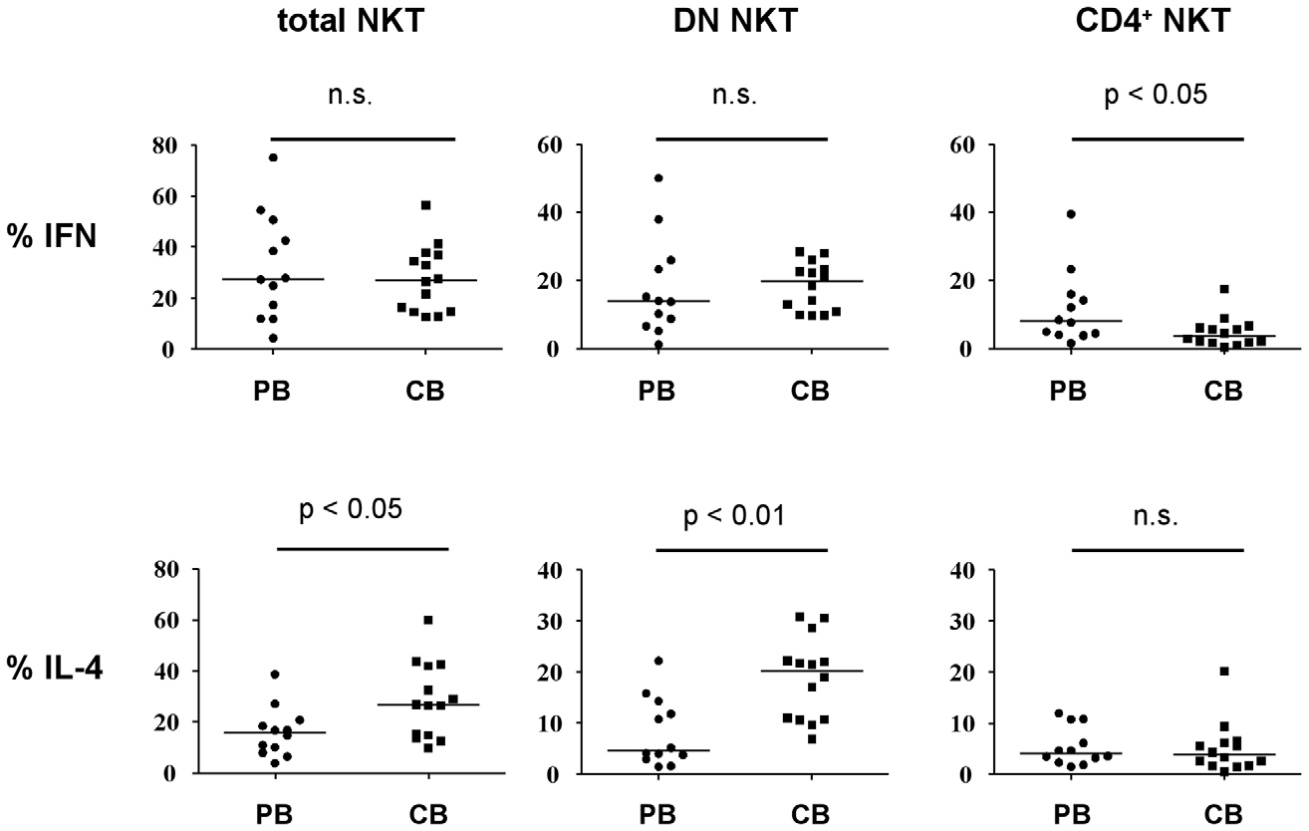
Percentages of IL-4 and IFN-γ secreting NKT cells within total, CD4^+^ and DN subsets. Horizontal bars indicate medians of percentages of IFN-γ (upper panel) and IL-4 (lower panel) secreting NKT cells within total (left), DN (middle) and CD4^+^ (right) NKT cell subsets. Cord blood (CB) NKT cells and peripheral blood (PB) NKT cells from adults are compared.

Since chemokine receptor analyses in total NKT cells indicated a Th2-bias in the cord blood NKT cells, we set up a second series of experiments to assess the cytokine production of total NKT cells and single CKR^+^ NKT subsets by six color flow cytometry. Cord blood total NKT cells produced IL-4 at significantly higher frequencies than peripheral NKT cell, whereas production of IFN-γ was similar (**Figure 1** **and** **2**). Since this pointed to a Th2-bias in total NKT cells intracellular staining was performed additionally for IL-13 and IL-10. The frequency of IL-10 secreting NKT cells was highly variable in cord blood (n = 3: 0%; 2.1% and 33%) and barely detectable in NKT cells from adults (n = 3: 0%; 1.3%; 1.5%). The frequency of IL-13 secreting NKT cells was higher in cord blood (1.3%, 13.1%, 14.3%; versus 1.06%, 2.17%, 2.6%) thereby supporting the presence of a Th2-bias in cord blood NKT cells.

**Figure 2:**
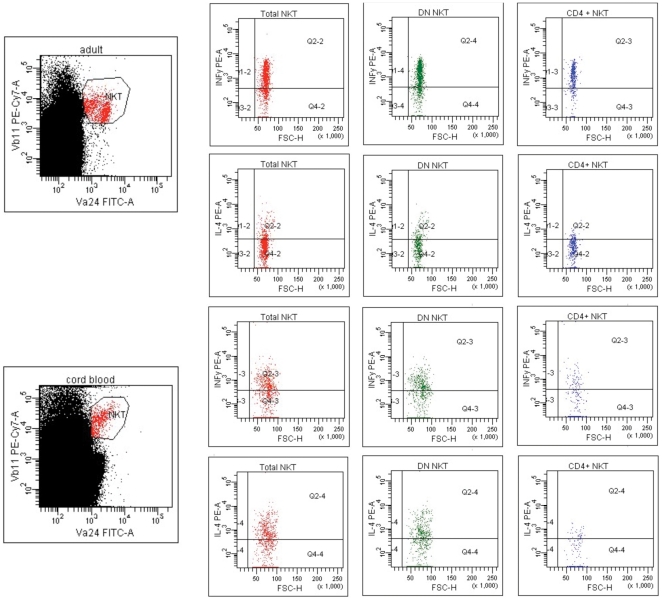
Representative dot-plots of IL-4 and IFN-γ secreting NKT cells within total, CD4^+^ and DN subsets. NKT cells from adult peripheral blood (upper two panels) and cord blood NKT cells (lower two panels) are compared. NKT cells were first identified by double positive staining of Vα24 and Vβ11 TCR chains (left). IFN-γ and IL-4 secreting NKT cells are shown within total (left), DN (middle) and CD4^+^ (right) NKT cell subsets.

A further subanalysis of the major NKT subpopulations showed that the frequency of IFN-γ producing cells tended to be lower in the cord blood DN NKT population, while the frequency of IL-4 producing NKT cells was significantly higher compared to NKT cells from adults. Among the CD4^+^ NKT cells the frequency of IFN-γ producing cord blood NKT was significantly lower (p = 0.048) compared to NKT cells from adults, while the frequency of IL-4 producing cells did not differ. Thus, overall there was a Th2-bias in the dominant NKT subpopulations in cord blood.

As expected, PMA/ionomycin stimulation led to a marked downregulation of surface CKR expression; yet it was possible to identify CKR expressing NKT cell subsets in sufficient numbers for analyses.

Among DN NKT cells, the frequency of IL-4 producing cells was significantly higher within CCR3^+^, CCR4^+^, CCR5^+^, CCR6^+^, CCR7^+^, CCR8^+^ and CXCR4^+^ subsets in cord blood compared to their peripheral blood counterparts from adults (**Table 2**). Within DN NKT cells, a higher frequency of IFN-γ producing cells could be detected in CCR4^+^, CCR7^+^ and CXCR4^+^ cord blood NKT cells. Among the CD4^+^subsets, IL-4 secreting cells were more frequent in CXCR3^+^ NKT cells from adults. This resulted in significantly lower IFN-γ/IL-4 ratios for CCR3^+^ (p = 0.01), CCR6^+^ (p<0.001) and CCR8^+^ (p = 0.03) DN NKT cells and also for CCR6^+^ CD4^+^ NKT cells (p = 0.05) in cord blood.

**Table 2:** Percentages of IL-4 and IFN-*γ* secreting NKT cells within NKT subsets.

			Cord blood (n = 14)	Peripheral blood (n = 12)	p-value
**CCR2**	DN	IFN	1.00^a^ (0.10–4.17)	0.31 (0.12–4.10)	0.07
		IL-4	1.23 (0.00–5.83)	0.28 (0.00–3.35)	0.12
	CD4	IFN	0.40 (0.00–2.34)	0.33 (0.10–1.61)	0.43
		IL-4	0.28 (0.00–1.73)	0.16 (0.00–0.60)	0.49
**CCR3**	DN	IFN	1.04 (0.10–4.58)	0.43 (0.00–1.03)	0.19
		IL-4	0.95 (0.00–6.81)	0.17 (0.00–1.53)	**<0.02**
	CD4	IFN	0.39 (0.00–1.54)	0.46 (0.09–3.34)	0.27
		IL-4	0.22 (0.00–0.78)	0.18 (0.00–0.73)	0.70
**CCR4**	DN	IFN	2.30 (0.31–6.47)	0.52 (0.00–2.34)	**0.01**
		IL-4	2.17 (0.37–12.56)	0.21 (0.00–1.55)	**<0.001**
	CD4	IFN	0.60 (0.00–2.10)	0.50 (0.00–1.65)	0.20
		IL-4	0.52 (0.00–11.07)	0.29 (0.00–1.01)	0.09
**CCR5**	DN	IFN	0.65 (0.05–3.72)	0.29 (0.23–1.21)	0.66
		IL-4	0.89 (0.20–4.66)	0.19 (0.00–1.75)	**<0.004**
	CD4	IFN	0.30 (0.00–1.13)	0.32 (0.00–1.83)	0.50
		IL-4	0.25 (0.00–2.00)	0.27 (0.00–0.77)	0.86
**CCR6**	DN	IFN	1.84 (0.10–8.59)	0.91 (0.45–4.07)	0.08
		IL-4	2.16 (0.43–14.12)	0.52 (0.13–3.66)	**<0.01**
	CD4	IFN	0.28 (0.00–2.14)	0.42 (0.17–2.47)	0.15
		IL-4	0.60 (0.00–2.83)	0.45 (0.12–1.46)	0.85
**CCR7**	DN	IFN	0.34 (0.10–2.56)	0.06 (0.00–0.65)	**<0.01**
		IL-4	0.43 (0.00–2.70)	0.15 (0.00–1.45)	**<0.02**
	CD4	IFN	0.16 (0.00–3.00)	0.16 (0.00–1.42)	0.80
		IL-4	0.28 (0.00–0.66)	0.10 (0.00–0.72)	0.19
**CCR8**	DN	IFN	0.87 (0.14–2.80)	0.43 (0.00–3.17)	0.11
		IL-4	0.94 (0.40–5.29)	0.27 (0.00–1.82)	**<0.01**
	CD4	IFN	0.16 (0.00–1.07)	0.30 (0.00–1.18)	0.14
		IL-4	0.16 (0.00–2.07)	0.03 (0.00–0.60)	0.20
**CCR9**	DN	IFN	1.74 (0.00–9.15)	0.88 (0.37–5.25)	0.25
		IL-4	1.82 (0.14–9.86)	0.92 (0.19–6.27)	0.14
	CD4	IFN	0.22 (0.00–1.02)	0.39 (0.00–0.88)	0.12
		IL-4	0.17 (0.00–3.58)	0.40 (0.00–1.65)	0.19
**CXCR3**	DN	IFN	1.23 (0.08–6.54)	0.45 (0.00–1.54)	0.11
		IL-4	1.01 (0.00–6.13)	0.63 (0.11–1.86)	0.16
	CD4	IFN	0.32 (0.00–1.53)	0.58 (0.06–2.11)	0.06
		IL-4	0.10 (0.00–3.64)	0.50 (0.16–1.17)	**0.02**
**CXCR4**	DN	IFN	2.33 (0.67–13.82)	0.93 (0.00–6.46)	**0.01**
		IL-4	3.29 (1.06–15.15)	0.76 (0.00–7.73)	**<0.01**
	CD4	IFN	0.23 (0.00–2.52)	0.26 (0.00–1.29)	0.98
		IL-4	0.18 (0.00–2.86)	0.20 (0.00–1.65)	0.64

a Data are shown as medians with (min-max.).

### Confirmation of invariant Vα24^+^ chain in cord blood

To rule out the possibility that cord blood Vα24^+^Vβ11^+^ T cells represent an unusual subset of conventional T cells with non-invariant TCR alpha, we sorted Vα24^+^Vβ11^+^ T cells from six cord blood samples and from adult peripheral blood. Sequencing of AV24-AJ18 transcripts showed the characteristic rearrangement without N-additions also in cord blood NKT cells. Further sorting of NKT cells into DN, CD4^+^ and CD8^+^ populations confirmed again the absence of N-additions in cord blood Vα24^+^Vβ11^+^ CD4^+^ NKT cells (**Table 3**). Due to the low frequency of DN and CD8^+^ cord blood NKT cells it was not possible sort sufficient cells for sequencing.

**Table 3:** Sequences of sorted fetal and non-fetal NKT cell populations.

		AV24	N	AJ18
**CanonicalAV24-AJ18 sequence**		**tac atc tgt gtg gtg agc**		**gac aga ggc tca acc ctg**
**Adult donors**				
Donor 1	DN	tac atc tgt gtg gtg agc		gac aga ggc tca acc ctg
	CD4^+^	tac atc tgt gtg gtg agc		gac aga ggc tca acc ctg
	CD8^+^	tac atc tgt gtg gtg agc		gac aga ggc tca acc ctg
Donor 2	DN	tac atc tgt gtg gtg agc		gac aga ggc tca acc ctg
	CD4^+^			
	CD8^+^			
Donor 3	DN	tac atc tgt gtg gtg agc		gac aga ggc tca acc ctg
	CD4^+^	tac atc tgt gtg gtg agc		gac aga ggc tca acc ctg
	CD8^+^	tac atc tgt gtg gtg agc		gac aga ggc tca acc ctg
**Cord blood samples**				
Sample 1	CD4^+^	tac atc tgt gtg gtg agc		gac aga ggc tca acc ctg
Sample 2	CD4^+^	tac atc tgt gtg gtg agc		gac aga ggc tca acc ctg
Sample 3	CD4^+^	tac atc tgt gtg gtg agc		gac aga ggc tca acc ctg

## Discussion

The present study shows that human cord blood Vα24^+^Vβ11^+^ NKT exhibit a strong bias towards Th2-responses which is restricted to specific NKT subsets which are distinguished by the expression of chemokine receptors and the absence of CD4.

NKT cells were defined by the coexpression of Vα24 and Vβ11 TCR chains. We considered this definition as most suitable for cord blood NKT cells, since CD161 is expressed at low levels in cord blood NKT cells [20], [21] (and own observations). Further, conventional T cells can also express CD161 upon stimulation [10], [22]. The definition of NKT cells by their responsiveness to glycolipids is also non-exclusive, because CD1d restricted cells that do not use Vα24 may also respond [23], [24], [25], [26]. Furthermore, our definition of NKT cells appears valid, since TCR sequencing of sorted cord blood NKT confirmed the typical invariant Vα24-Jα18 TCR chain without N-additions.

Similar to peripheral blood NKT cells from adults, cord blood NKT cells expressed a broad range of chemokine receptors that can direct migration both to lymphoid and non-lymphoid tissues. With the exception of CXCR5 our findings on peripheral blood NKT cells match those of Kim et al.[27] very closely. Peripheral blood NKT cells from adult donors have a predominant Th1/inflammatory chemokine receptor profile [4], [27], [28]. This is contrasted by higher frequencies of Th2-associated CCR4^+^ and CCR8^+^ NKT cells and lower frequencies of Th1-related CCR5^+^ and CXCR3^+^ NKT subsets among total NKT cells in cord blood as observed here. CXCR6^+^ NKT cells were more abundant in cord blood. CXCL6 is abundantly secreted by the fetal trophoblast [29], indicating recruitment of cord blood NKT cells to the materno-fetal interface. Our study further shows that the frequencies of CCR7 and CXCR5 expressing NKT cells are higher in cord blood. This is in line with earlier reports of their low frequencies in adult peripheral blood [28], [30], as well as the high frequency of CD62L^+^ cord blood NKT cells [20] and indicates that cord blood NKT cells could home to lymph nodes and B cell areas, respectively. The simultaneous expression of markers typical for naïve cells and the memory marker CD45RO as observed by us and others [5], [6] with concomitant high CCR7 expression is intriguing. It has been shown earlier that the fetus is able to mount antigen specific immune responses to environmental antigens [31], [32]. Since the expression of CD45RO indicates previous antigen contact, we speculate that cord blood CD45RO^+^ NKT cells are functionally mature and migrate to lymphatic tissue to ensure tolerance during *in utero* exposure to antigens. Even though the chemokine receptor profile is obviously different between cord blood and peripheral NKT cells, is has not been studied if the chemokine receptors on cord blood NKT cells are functionally active and mediate cellular migration.

Because most chemokines are ligands for several receptors to define the functionality of specific receptors would involve migration assays with NKT cells sorted according to the select chemokine receptors. However, due to the limited amount of NKT cells in cord blood this approach is not feasible. Migration assays combined with blocking of specific chemokine/chemokine receptor interactions could help resolve this issue. Yet, due to the limited amount of NKT cells in cord blood this approach is highly challenging.

The low frequency of NKT cells further makes their accurate quantification difficult. Nonetheless, several facts support the reliability of our measurements: first, we used very high total numbers of lymphocytes for flow cytometry; second, the finding that the majority of cord blood NKT cells was CD4^+^ is in line with previous publications [20], [21], [30], [33]; third, higher frequencies of CCR7^+^ NKT cells in cord blood were also observed by others [20]. Contamination with maternal blood cannot be excluded with certainty based on the analysis of IgA in cord blood. However, due to the general paucity of NKT cells in blood a transfer of maternal NKT cells to the fetus in the quantities measured here seems unlikely. Furthermore, CD45RO expression on cord blood NKT cells has been reported also by others [5], [6].

The observed chemokine receptors expression pattern indicates Th2-biased functions of cord blood NKT cells which were further substantiated by the IL-4/IFN-γ secretion patterns of cord blood NKT cells. Within total NKT cells, IL-4 producing NKT cells were more frequent in cord blood compared to adult peripheral blood, in accordance with a previous report [34]. Within the DN subset the higher frequency of IL-4 production was not limited to NKT cells expressing Th2-associated CCR4 and CCR8, but occurred in the majority of DN NKT cell subsets. Of note, the stimulation protocol used here, rather reflects the general disposition of NKT cells to produce certain cytokines but does not give information whether they behave in the same manner *in utero* during normal pregnancies. Finally, a comparatively small number of newborns was analyzed which reduces the power to detect small but potentially important differences. Therefore, it is possible that some findings were by chance. On the other hand, the size of differences between cord blood and peripheral NKT cells was rather high which argues for their biological relevance. For this reason, it seems unlikely that the analysis of a larger sample size would have yielded substantially different results.

Based on the analysis of chemokine receptor expression patterns and IL-4/IFN-γ secretion, cord blood NKT cells constitute a heterogeneous population. Single NKT cell subsets might have different specialized immune regulatory functions during fetal life. Yet, despite this heterogeneity, we observed a clear Th2-bias mainly in the DN subset. Therefore, we speculate that NKT cells are important for the initiation and control of the cord blood Th2 environment which is needed to maintain tolerance towards self-antigens as well as non-inherited maternal antigens. Moreover, a NKT mediated Th2 bias may also be important in the early post-natal period to prevent overactive immune responses during early extrauterine exposures towards ubiquitous antigens.
